# Environmental Oxygen Tension Regulates the Energy Metabolism and Self-Renewal of Human Embryonic Stem Cells

**DOI:** 10.1371/journal.pone.0062507

**Published:** 2013-05-06

**Authors:** Catherine E. Forristal, David R. Christensen, Fay E. Chinnery, Raffaella Petruzzelli, Kate L. Parry, Tilman Sanchez-Elsner, Franchesca D. Houghton

**Affiliations:** Centre for Human Development, Stem Cells and Regeneration, Faculty of Medicine, University of Southampton, Southampton, United Kingdom; University of Newcastle upon Tyne, United Kingdom

## Abstract

Energy metabolism is intrinsic to cell viability but surprisingly has been little studied in human embryonic stem cells (hESCs). The current study aims to investigate the effect of environmental O_2_ tension on carbohydrate utilisation of hESCs. Highly pluripotent hESCs cultured at 5% O_2_ consumed significantly more glucose, less pyruvate and produced more lactate compared to those maintained at 20% O_2_. Moreover, hESCs cultured at atmospheric O_2_ levels expressed significantly less OCT4, SOX2 and NANOG than those maintained at 5% O_2_. To determine whether this difference in metabolism was a reflection of the pluripotent state, hESCs were cultured at 5% O_2_ in the absence of FGF2 for 16 hours leading to a significant reduction in the expression of SOX2. In addition, these cells consumed less glucose and produced significantly less lactate compared to those cultured in the presence of FGF2. hESCs maintained at 5% O_2_ were found to consume significantly less O_2_ than those cultured in the absence of FGF2, or at 20% O_2_. GLUT1 expression correlated with glucose consumption and using siRNA and chromatin immunoprecipitation was found to be directly regulated by hypoxia inducible factor (HIF)-2α at 5% O_2_. In conclusion, highly pluripotent cells associated with hypoxic culture consume low levels of O_2_, high levels of glucose and produce large amounts of lactate, while at atmospheric conditions glucose consumption and lactate production are reduced and there is an increase in oxidative metabolism. These data suggest that environmental O_2_ regulates energy metabolism and is intrinsic to the self-renewal of hESCs.

## Introduction

Human embryonic stem cells (hESCs) are pluripotent cells derived from the inner cell mass (ICM) of the blastocyst, the final stage of preimplantation embryo development. They proliferate through self-renewal and provide an excellent model to investigate developmental mechanisms since they have the potential to differentiate into all cells of the body [1]. However, if these cells are to be of therapeutic use it is imperative to ensure that a highly pluripotent population of hESCs are maintained which can then be directed down specific lineage pathways.

hESCs are notoriously difficult to maintain *in vitro* as the colonies have a propensity to spontaneously differentiate suggestive of suboptimal culture conditions; an effect which may be circumvented by the use of low environmental O_2_ tensions [2]. Culture under atmospheric O_2_ tensions has been found to decrease hESC proliferation and reduce pluripotency marker expression compared to culture under low (2–5%) O_2_ tensions [3], [4], [5], an effect regulated by hypoxia inducible factors (HIFs), specifically HIF-2α [5]. This promotion of an immature, stem cell like phenotype has also been observed in both malignant and non-malignant cells cultured under hypoxic conditions [6]. Despite these encouraging data, many of the biochemical and physiological implications of hypoxic culture on hESCs remain to be elucidated.

Among the ∼200 genes regulated by HIFs, metabolic genes feature extensively [7] suggesting that environmental O_2_ tensions may also have a significant impact on hESC metabolism. However, the metabolic status of hESCs and the impact of environmental O_2_ tension have received remarkably little attention. Significantly, our previous work has shown that metabolic activity is a central regulator of the phenotype and developmental potential of human preimplantation embryos [8], [9] and highlights metabolism as a fundamental regulator of cellular function.

Morphologically, hESCs share many characteristics with ICM cells; a high nuclear to cytoplasmic ratio, low mitochondrial number, and expression of the same surface antigens [10], [11], [12], [13]. It is therefore likely that the nutrition of the ICM may inform on the metabolism of hESCs. In terms of glucose utilisation, the murine ICM is wholly glycolytic compared to the differentiated trophectoderm where only 55% of the glucose consumed may be accounted for by lactate production [14]. Moreover, the ICM is also metabolically relatively quiescent in terms of mitochondrial activity, O_2_ consumption and ATP production compared to the trophectoderm [15]. Knowledge of the metabolism of hESCs is still in its infancy but it has been shown that pluripotency may be enhanced by inhibiting the mitochondrial respiratory chain [16]. This data was further supported by confirmation that hESCs and induced pluripotent stem cells rely on glycolysis for their energy requirements [17], [18], [40].

This study aims to investigate how environmental O_2_ tension affects the regulation and energy metabolism of hESCs in terms of O_2_, glucose and pyruvate consumption and lactate production. Moreover, the effect of early hESC differentiation as demonstrated by the short term removal of FGF2 on hESC metabolism and pluripotency marker expression has also been investigated. These data suggest that environmental O_2_ regulates glucose utilisation and is intrinsic to the energy metabolism and self-renewal of hESCs.

## Materials and Methods

### hESC Culture

Hues7 (D. Melton, Howard Hughes Medical Institute/Harvard University) [41] and Shef3 (Supplied by the UK Stem Cell Bank) hESCs were cultured at 20% O_2_ in Knockout DMEM (Invitrogen) supplemented with 15% knockout serum replacement (Invitrogen), 100 µg/ml penicillin streptomycin (Invitrogen), 1 mM L-glutamine (Invitrogen), 1× non-essential amino acids (Invitrogen), 0.1 mM 2-mercaptoethanol and 10 ng/ml FGF2 (Peprotech) on γ irradiated mouse embryonic fibroblasts (MEFs; a primary source derived in institutional facilities following University of Southampton ethical review committee approval and in accordance with UK Home Office regulations). hESCs were then transferred to Matrigel (BD Biosciences) coated plates and cultured in MEF-conditioned medium at both 20% and 5% O_2_. They were maintained for a minimum of 3 passages on Matrigel at both O_2_ tensions prior to use.

### Measurement of Carbohydrate Utilisation

hESCs were passaged on to 12-well Matrigel coated plates and cultured in MEF conditioned medium. On day 2, 3 and 4 post-passage hESCs were pre-incubated in a defined metabolic medium [8] containing 1 mM glucose, 5 mM lactate, 0.47 mM pyruvate, 0.5% human serum albumin and amino acids [19] for 30 mins. The medium was then replaced with pre-determined quantities (300–500 µl) of defined medium for 1.5–3.5 h. At the end of the incubation period, all but 100 µl of medium was removed from each well and stored at −80°C prior to analysis of carbohydrate content and the number of cells in each well was determined using a haemocytometer. In subsequent experiments, the effect of removing FGF2 for 16 hours on hESC metabolism was monitored. FGF2 was removed from the medium prior to MEF conditioning and used to replace regular, MEF conditioned medium containing FGF2 on day 2 post-passage. Enzyme linked biochemical assays were used to measure the concentration of pyruvate, glucose and lactate in 180 µl of spent medium using a Konelab 20 autoanalyser (Thermo Scientific). The concentration of carbohydrates in cell containing wells was compared to cell-free control wells and the consumption of pyruvate and glucose and the production of lactate by hESCs calculated in pmol/cell/h.

### O_2_ Assay

A 96-well O_2_ biosensor plate (BD Biosciences) containing 3 wells of 200 mM sodium sulphite (0% O_2_ control) and 3 wells of a defined metabolic medium (20% O_2_ control) was incubated at 37°C in a fluorescence plate reader (BMG Labtech) for 30 mins. Hues7 hESCs on day 3 post-passage were pre-incubated with metabolic medium for 30 mins, harvested into 310 µl of fresh, pre-warmed metabolic medium, added to a well of the O_2_ biosensor plate and sealed using an adhesive PCR foil (Thermo Fisher) with care taken to ensure the absence of air bubbles. The fluorescence (excitation 485 nm and emission 612 nm) of each well was recorded every 2 minutes over a two hour period. After the final measurement, the protein content of each well was determined using the Bradford assay. O_2_ consumption was calculated as µl O_2_/mg protein/h.

### RT-qPCR

RNA was isolated from Hues7 hESCs cultured under feeder-free conditions on Matrigel on day 3 post-passage using TriReagent (Sigma) and 2 µg reverse transcribed to cDNA using MMLV-reverse transcriptase (Promega). cDNA (4 µg) was amplified in 20 µl reactions containing 1 µl probes and primer mix (OCT4: Hs01895061_u1; SOX2: Hs00602736_s1; NANOG: Hs02387400_g1; GLUT1: Hs00197884_m1; UBC Hs00824723_m1) and 10 µl 2× Taqman Universal PCR Master Mix (Applied Biosystems) using an ABI 7500 real time PCR system. The conditions used were 2 mins at 50°C, 10 mins at 95°C followed by 45 cycles of 95°C for 15 secs and 60°C for 1 min. Placental cDNA (0–10 ng) was used to produce a standard curve for each gene of interest as well as the endogenous control, UBC and used to quantify gene expression. All genes were analysed in duplicate and normalised to UBC.

### siRNA

siRNA was used to silence either *HIF-1α*, *HIF-2α* or *HIF-3α* in Hues7 hESCs cultured on Matrigel coated plates at 5% O_2_. The cells were passaged and the following day 50 nM siRNA (HIF-1α: Hs_HIF1A_5; HIF-2α: Hs_EPAS1_5; or HIF-3α: Hs_HIF3A_1), 12 µl HiPerfect transfection reagent (Qiagen) and 200 µl knockout DMEM were mixed, incubated at room temperature for 10 mins and added in a drop wise manner to hESCs. Allstars negative control siRNA (Qiagen) was used as a negative control. The ability of these siRNA to silence individual HIF-α isoforms has previously been validated [5]. The cells were harvested 48 h post-transfection and *GLUT1* mRNA quantified as above.

### Western Blotting

Protein was isolated from Hues7 hESCs cultured on Matrigel on day 3 post-passage by incubating in ice cold radio immunoprecipitation assay (RIPA) buffer for 30 mins followed by sonication for 30 secs. Protein (75 µg) was resolved on an 8% SDS bisacrylamide gel, transferred to nitrocellulose membrane and blocked in PBS containing 0.1% Tween-20 and 5% milk for 1 h at room temperature. The membrane was incubated in primary antibody (OCT4 (Santa Cruz) 1∶1000; SOX2 (Millipore) 1∶1000; NANOG (Abcam) 1∶1000) diluted in blocking buffer overnight at 4°C. Membranes were washed and incubated in horse radish peroxidase-conjugated secondary antibodies (anti-mouse (GE Healthcare) 1∶100,000 or anti-rabbit (GE Healthcare) 1∶50,000) for 1 h at room temperature. The Enhanced chemiluminescence advanced Western blotting detection kit (GE Healthcare) was used to develop the membranes prior to imaging on the Biorad Chemidoc XRS. Protein expression was quantified relative to β-actin (mouse anti-β-actin peroxidise conjugated antibody (Sigma) 1∶50,000).

### Chromatin Immunoprecipitation (ChIP) Assays

Hues7 hESCs cultured in normoxic (20% O_2_), or hypoxic (5% O_2_) conditions were cross-linked with 1% formaldehyde for 10 min and the reaction blocked with 0.125 M glycine. ChIP experiments were performed with HIF-2α (Novus Biologicals) or immunoglobulin G (IgG) antibody (Santa Cruz) as previously described [20], [21] except that immuno-complexes were washed using high-salt buffers as followed: 10 times with 600 µl buffer A (0.1% SDS, 2 mM EDTA, 20 mM Tris HCl pH8, 1% Triton X-100, 500 mM NaCl), 8 times with 600 µl buffer B (0.1% SDS, 2 mM EDTA, 20 mM Tris HCl pH8, 1% Triton X-100, 1 M NaCl), 3 times with 600 µl TE (10 mM Tris HCl H8, 1 mM EDTA) buffer. Recovered DNA was amplified with custom Taqman Assays (Applied Biosystems) spanning a predicted hypoxia response element (HRE) site at −1691 bp of the GLUT1 proximal promoter (GLUT1 fwd: CAAATGTGTGGATGTGAGTTGC; GLUT1 rev: CCATCACGGTCCTTCTTCATG; GLUT1 probe: AGGCTGAGCGTGTAAA). qPCR was performed using an ABI 7900 HT Fast Real Time System (Applied Biosystems) in a 384 well plate.

### Statistical Analysis

All data were tested to determine whether they were normally distributed using the Anderson Darling normality test. Any differences in the utilisation of carbohydrates or glycolytic rate with O_2_ tension were analysed using a Student’s t-test. Differences in mRNA expression were normalised to the endogenous control, UBC and then to 1 for cells cultured at 5% O_2_, or to Allstars transfection controls when genes were silenced. Differences in protein expression were normalised to β-actin and then to 1 for cells cultured at 5% O_2_. In both cases a one sample t-test was used to determine significance from either 5% O_2_ or transfection controls. Differences in O_2_ consumption between hESCs maintained at 5%, 20% and 5% O_2_ in the absence of FGF2 was determined using a one-way analysis of variance followed by a Fisher’s test. Differences in binding of HIF-2α to the GLUT1 promoter with O_2_ tension was expressed as a percentage of input (non-immunoprecipitated chromatin) calculated using 100×2^[Ct (Input)-Ct (IP)]^ for each sample and expressed as box and whisker plots. All data represent at least 3 independent experiments and are presented as mean ± SEM.

## Results

### Environmental O_2_ Tension Regulates Carbohydrate Utilisation

**Figure 1:**
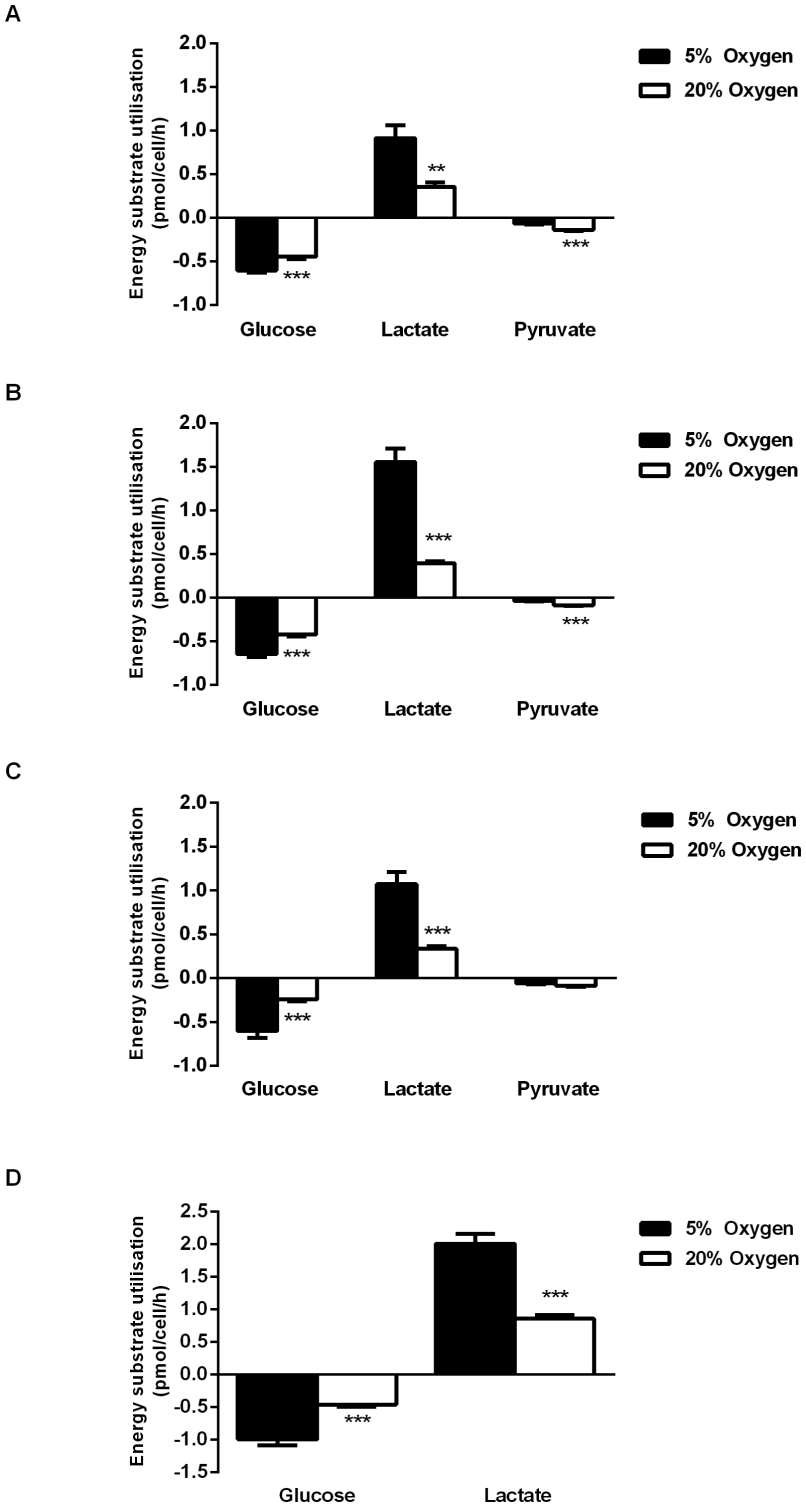
Hypoxic culture promotes glucose uptake and lactate production in hESCs. Glucose, pyruvate and lactate utilisation were non-invasively measured in a defined hESC medium. More glucose was consumed and lactate produced by Hues7 hESCs cultured at 5% O_2_ than at 20% O_2_ on (A) day 2 (B) day 3 and (C) day 4 post-passage. In contrast, less pyruvate was consumed by hESCs at 5% O_2_ compared to 20% O_2_. The rate of glucose consumption and lactate production was also greater on day 3 post-passage in Shef3 hESCs cultured at 5% O_2_ compared to those maintained at 20% O_2_ (D). **P<0.01, ***P<0.001 significantly different to 5% O_2_ (n = 12–23).

hESCs maintained at 5% O_2_ display an increased proliferation and expression of pluripotency markers compared to those cultured at 20% O_2_ [5]. The current study aimed to determine the impact of environmental O_2_ tension on the energy metabolism of hESCs. Hues7 hESCs were cultured at either 5% or 20% O_2_ and the consumption of glucose and pyruvate and the production of lactate were analysed on days two to four post-passage. Similarly, the effect of environmental O_2_ tension on the depletion of glucose and production of lactate by Shef3 hESCs on day 3 post-passage was also determined. Under each O_2_ tension, glucose was found to be the predominant energy substrate utilised. Interestingly, both cell lines consumed almost twice as much glucose (P<0.001) and produced approximately 2–3 times the amount of lactate (P<0.01–P<0.001) when cultured at 5% O_2_ compared to 20% O_2_ (Fig. 1A–D). A similar, low level (∼0.1 pmol/cell/h) of pyruvate was consumed by Hues7 hESCs on each day post-passage (Fig. 1 A–C).

### Environmental O_2_ and Short-term Removal of FGF2 Alters hESC Energy Metabolism

To investigate whether the difference in energy substrate utilisation between hESCs cultured at 5% and 20% O_2_ was a reflection of the degree of cell pluripotency, FGF2 was removed from the medium used to culture cells at 5% O_2_ for 16 hours. Morphologically, there was no overt differentiation observed in any of the treatment groups on day 3 post-passage (Fig. S1). This is in agreement with previous observations of hESCs cultured at 5% or 20% O_2_ [5].

**Figure 2:**
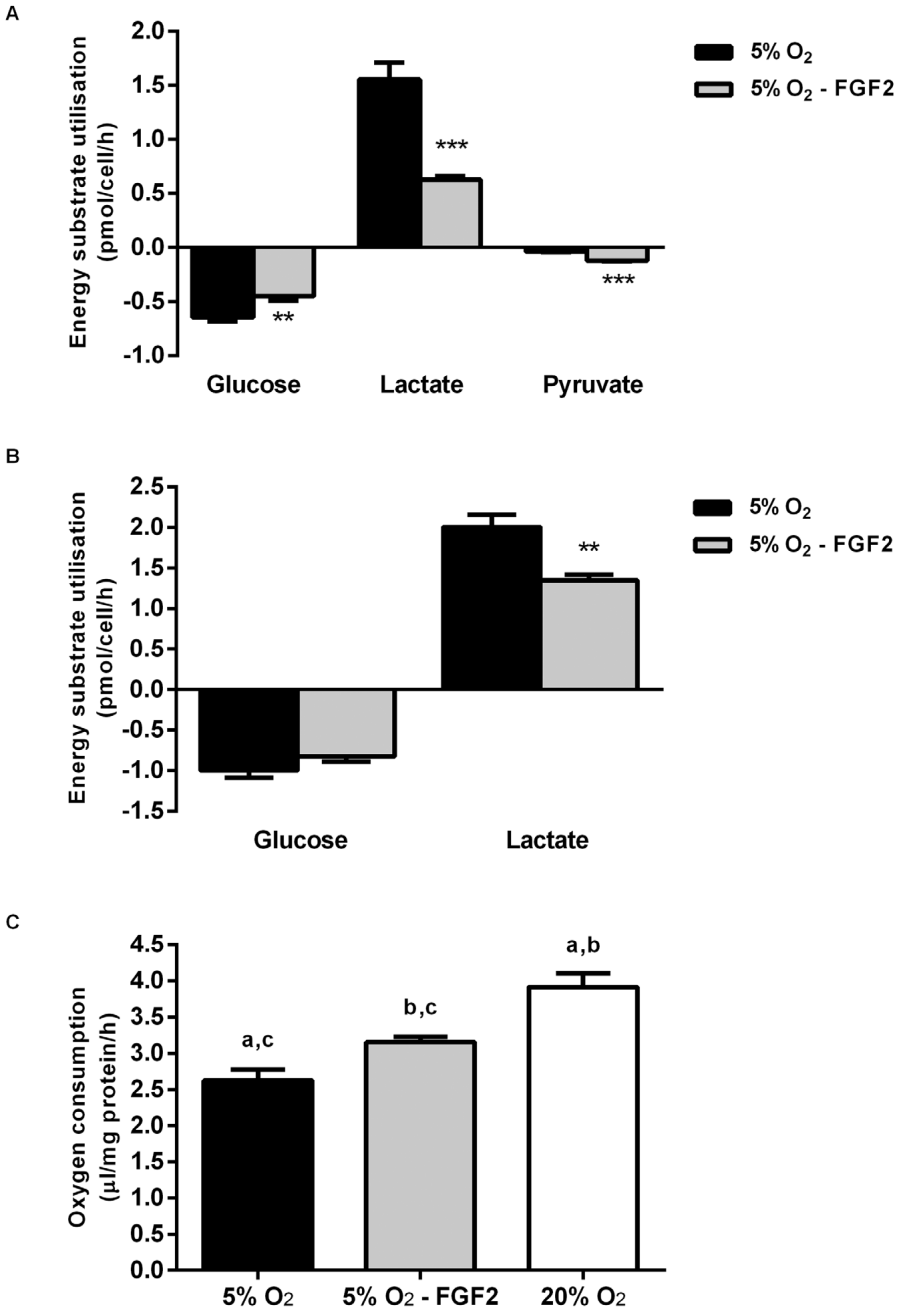
Short term removal of FGF2 at 5% O_2_ alters hESC metabolism and promotes O_2_ consumption. Removal of FGF2 for 16 hours from Hues7 hESCs cultured at 5% O_2_ (5% O_2_– FGF2) resulted in a reduction of glucose consumption and lactate production, whereas pyruvate consumption dramatically increased (A). Shef3 hESCs cultured at 5% O_2_– FGF2 displayed a significant reduction in lactate production (B). **P<0.01, ***P<0.001 significantly different to 5% O_2_+FGF2 (n = 10–18). Hues7 hESCs cultured at 5% O_2_ consumed less O_2_ than when FGF2 was removed for 16 hours (C). hESCs maintained at 20% O_2_ consumed the greatest amount of O_2_. Bars with the same superscript are significantly different; a, b, P<0.001, c, P<0.05 (n = 7–8).

In terms of metabolism, removing FGF2 for 16 hours from hESCs cultured at 5% O_2_ resulted in a significant reduction in the amount of glucose consumed in Hues7 cells and a near significant reduction in Shef3 cells compared to those maintained in the presence of FGF2 (Fig. 2A and B). However, in the absence of FGF2, both cell lines displayed a significant reduction in the amount of lactate produced. Interestingly, twice as much pyruvate was consumed when Hues7 cells were cultured at 5% O_2_ in the absence of FGF2 for 16 hours compared to when FGF2 was present (P<0.01; Fig. 2 A). This suggests that even the very early stages of differentiation are associated with an increased reliance on oxidative metabolism.

To determine the global ability of hESCs to produce energy, O_2_ consumption was measured. Hues7 hESCs cultured at 20% O_2_ were found to consume approximately 4 µl O_2_/mg protein/h, which was significantly greater than hESCs cultured at both 5% O_2_ (P<0.001) and 5% O_2_ where FGF2 was removed for 16 hours (P<0.001; Fig. 2 C). Interestingly, the removal of FGF2 for 16 hours from hESCs cultured at 5% O_2_ significantly increased O_2_ consumption compared to those cultured in the presence of FGF2 (P<0.05). This suggests that hESCs maintained at 20% O_2_ have a greater energy requirement than those cultured at 5% O_2_ in the absence of FGF2. hESCs cultured at 5% O_2_ in the presence of FGF2 are the quietest metabolically having the lowest rate of O_2_ consumption.

### Environmental O_2_ and Short Term FGF2 Removal Regulates the Self-renewal of hESCs

**Figure 3:**
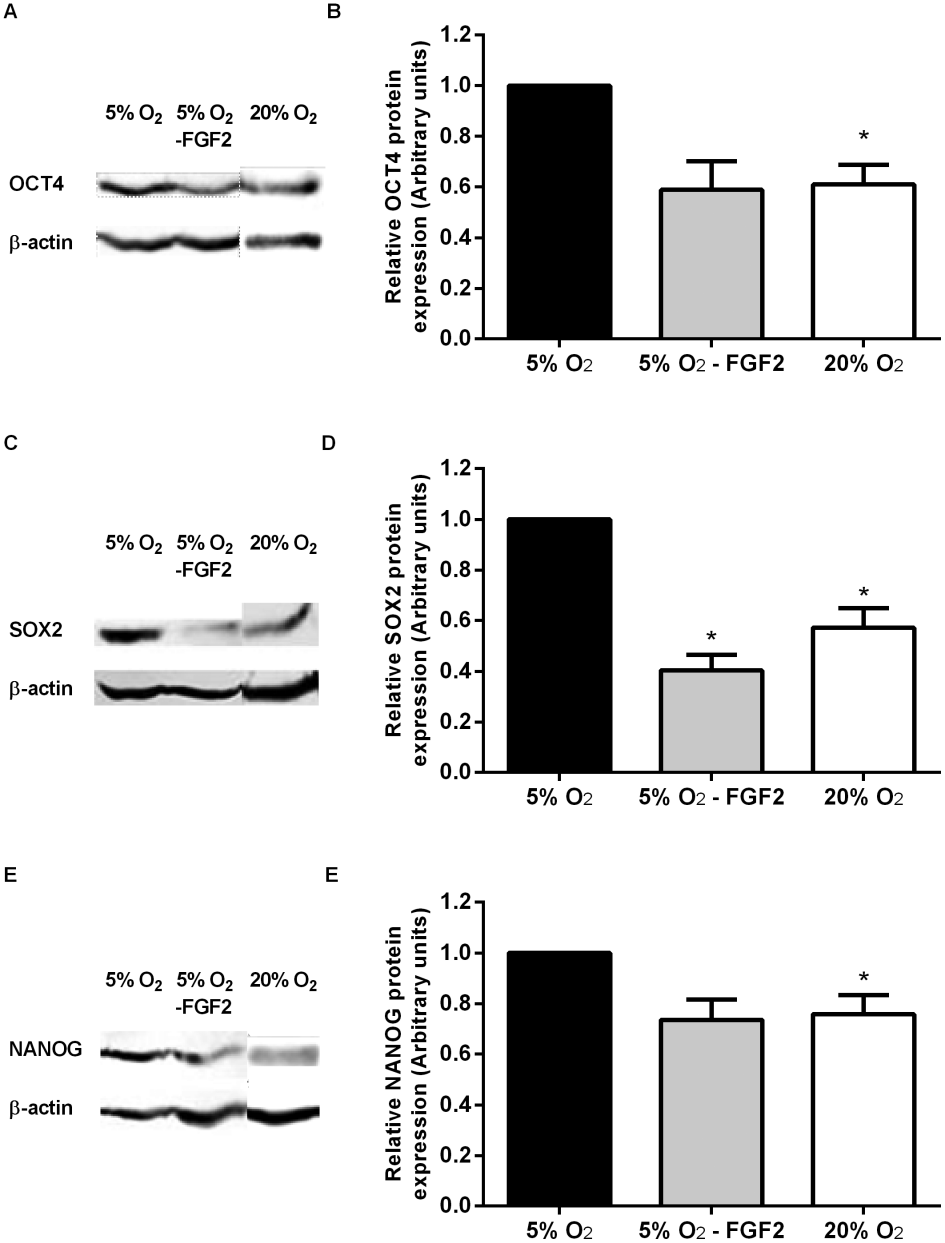
hESCs maintained at atmospheric O_2_ levels express reduced levels of pluripotency markers compared to those cultured at 5% O_2_. Hues7 hESCs were cultured at either 5% O_2_, 5% O_2_ with FGF2 removed for 16 hours (5% O_2_– FGF2) or 20% O_2_. Protein was isolated and OCT4 (A and B), SOX2 (C and D) and NANOG (E and F) quantified using Western blotting. All data has been normalised to β-actin and to 1 for 5% O_2_. *P<0.05, **P<0.01, ***P<0.001 significantly different from 5% O_2_ (n = 3–4).

In agreement with our previous report [5], OCT4 protein expression was significantly decreased in Hues7 hESCs maintained at 20% O_2_ compared to those cultured at 5% O_2_ (P<0.05; Fig. 3 A, B). A similar reduction in SOX2 (P<0.05) and NANOG (P<0.05) expression was also observed at 20% O_2_ (Fig. 3 C–F). Interestingly, when FGF2 was removed for 16 hours from hESCs cultured at 5% O_2_, SOX2 protein expression decreased significantly while OCT4 and NANOG expression displayed a non-significant reduction to levels comparable to hESCs cultured under atmospheric O_2_ tensions (Fig. 3A–F).

### GLUT1 mRNA is Differentially Expressed Under Hypoxic Conditions and Regulated by HIF-2α

**Figure 4:**
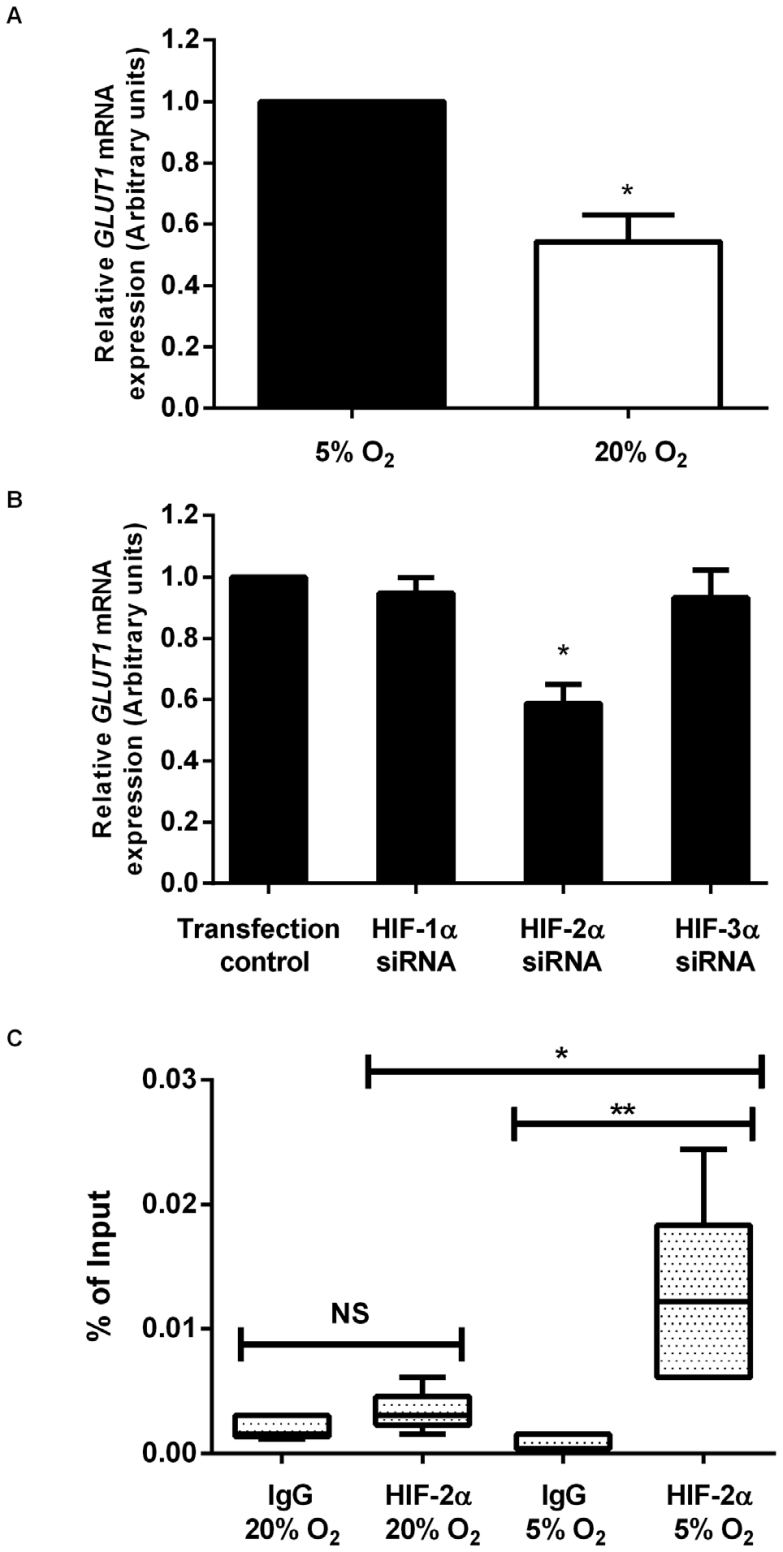
GLUT1 expression parallels glucose utilisation and is directly regulated by HIF-2α under hypoxic conditions. RT-qPCR was used to quantify *GLUT1* mRNA expression in Hues7 hESCs cultured at either 5% O_2_, or 20% O_2_ on day three post-passage (A). All data has been normalised to *UBC* and to 1 for 5% O_2_. *P<0.05 significantly different to 5% O_2_ (n = 3). Using siRNA to silence HIF-α subunits in Hues7 hESCs cultured at 5% O_2_, *GLUT1* mRNA was found to be regulated by HIF-2α (B). All data has been normalised to *UBC* and to 1 for the transfection control. *P<0.05 significantly different to transfection control (n = 6). Using ChIP HIF-2α was found to bind to the proximal promoter of GLUT1 only in hESCs cultured at 5% O_2_. ChIP assays were performed with either a HIF-2α or IgG control antibody on chromatin isolated from Hues7 hESCs cultured at either 20% O_2_ or 5% O_2_. DNA enrichment is expressed as a percentage of input (non-immunoprecipitated chromatin). *P<0.05, **P<0.01, NS indicates no significant difference (n = 5).

To determine whether differences in glucose transport may be responsible for the increased consumption observed by Hues7 hESCs maintained at 5% O_2_ compared to 20% O_2_, the expression of GLUT1 was investigated. GLUT1 mRNA expression was significantly decreased in cells maintained at 20% O_2_ compared to those cultured at 5% O_2_ (Fig. 4 A). This suggests a correlation between the mRNA expression of GLUT1 and the uptake of glucose in hESCs.

Since HIFs are important regulators of the hypoxic response, siRNA was used to determine whether any of the HIFα subunits were responsible for the increased GLUT1 expression in hESCs cultured at 5% O_2_. GLUT1 mRNA expression was not affected when either HIF-1α or HIF-3α were silenced but was significantly reduced when HIF-2α was knocked down (P<0.001; Fig. 4 B). This suggests that HIF-2α is an upstream regulator of GLUT1 in hESCs cultured at 5% O_2._

### HIF-2α Binds Directly to the GLUT1 Promoter

To determine whether HIF-2α binds directly to a potential HRE in the proximal promoter of GLUT1 ChIP assays were performed on Hues7 hESCs. We compared the enrichment, using qPCR, of the sequence corresponding to the GLUT1 proximal promoter when precipitating with an antibody specific for HIF-2α, compared to the IgG isotype control. A 4-fold enrichment over the IgG control was observed in the chromatin isolated from hESCs maintained at 5% O_2_ (Fig. 4C). In contrast, no significant binding of HIF-2α was observed in chromatin isolated from hESCs maintained at 20% O_2_. This data reveals a specific HIF-2α interaction with the GLUT1 proximal promoter only in hESCs cultured under hypoxic conditions.

## Discussion

hESC metabolism has received little attention, despite being intrinsic to cellular function. Several studies have highlighted beneficial effects of culturing hESCs at low O_2_ tensions including improved morphology, increased expression of pluripotency markers, a reduction in chromosomal abnormalities and a higher rate of proliferation [3], [4], [5], [22] but the impact on cellular metabolism is unknown. Thus, this study sought to investigate the influence of environmental O_2_ on the carbohydrate utilisation, energy metabolism and self-renewal of hESCs.

Independent of O_2_ tension, glucose was found to be the predominant substrate utilised by hESCs. However, highly pluripotent hESCs cultured under hypoxic conditions were found to deplete significantly more glucose and produce higher levels of lactate than cells maintained at atmospheric O_2_ tensions. This was intriguing and suggested a correlation between metabolism and self-renewal. To investigate this further, FGF2, a factor required to sustain self-renewal and support growth of undifferentiated hESCs [23], [24], was removed for just 16 hours from highly pluripotent cells cultured at 5% O_2_. The removal of FGF2 resulted in a reduced utilisation of glucose and significant decrease in the amount of lactate produced. This was intriguing and highlights the ability of hESC metabolism to adapt to changes in environmental conditions. Moreover, the resultant rates of glucose utilisation and lactate production observed in hESCs cultured in the absence of FGF2 for 16 hours were similar to cells cultured at 20% O_2_. This was interesting since cells maintained at 20% O_2_ expressed significantly less OCT4, SOX2 and NANOG than those cultured under hypoxic conditions. A similar trend was also mirrored by hESCs cultured at 5% O_2_ in the absence of FGF2. These data suggest that energy metabolism may represent a novel parameter to quantify the self-renewal potential of hESC cultures.

The mechanism of how FGF2 regulates hESC energy metabolism under hypoxic conditions is unknown but data from adipocytes implicates the involvement of HIF-1α [25]. These investigators found that the culture of adipocytes under hypoxic conditions in the presence of FGF2 caused an increase in both GLUT1 expression and lactate production through the induction of HIF-1α. In hESCs HIF-1α is degraded after ∼48 h of hypoxic culture after which HIF-2α is stabilised [5]. Thus, it could be speculated that the reduced amount of glucose consumed and lactate produced by hESCs cultured at 5% O_2_ in the absence of FGF2 may be due to the destabilisation/degradation of HIF-2α and the resultant decrease in expression of hypoxia responsive genes.

Hues7 hESCs cultured at 5% O_2_ consumed significantly lower levels of O_2_ and pyruvate than those maintained at either 20% O_2_ or 5% O_2_ in the absence of FGF2. Since O_2_ consumption provides the best global indication of the ability of a cell to produce energy, this suggests that hESCs cultured at 5% O_2_ are more metabolically quiescent than those cultured at 5% O_2_ in the absence of FGF2, or at 20% O_2_. As OCT4, SOX2 and NANOG expression were also significantly reduced in hESCs cultured at 20% O_2_ compared to 5% O_2_, this suggests that as differentiation occurs a more active metabolism ensues. These results are comparable to that in the mouse blastocyst where the ICM was found to be metabolically relatively quiescent consuming low levels of O_2_ compared to the differentiated trophectoderm [15].

Our data also suggest that hESCs display a glycolytic metabolism consuming glucose and producing lactate. This is in agreement with mouse ES cells and mesenchymal stem cells which utilise glycolysis as a primary source of ATP production in the undifferentiated state and switch to oxidative phosphorylation upon differentiation [26], [27]. Similarly, nuclear reprogramming associated with induced pluripotent stem cells has been shown to be associated with a shift from an oxidative metabolism to one dependent on glycolysis [18], [39]. Together with the current data, this highlights the importance of glycolysis for maintaining the pluripotent state.

hESCs cultured at 5% O_2_ expressed significantly more *GLUT1* than those maintained at 20% O_2_. Glucose transporter expression is known to increase the amount of glucose taken up by cells and GLUT1 is thought to be the predominant transporter in many cell types including mouse ESCs, brain, placenta, and retina [28], [29], [30]. Our finding of an increase in GLUT1 expression under hypoxic conditions is in agreement with that observed in mouse ESCs [31]. As a HRE is present in the promoter region of the GLUT1 gene [32], [33] we were interested to determine whether any of the 3 regulated HIF-α subunits were responsible for this increased expression. Using siRNA, HIF-α subunits were silenced individually and the effect on GLUT1 expression determined. GLUT1 mRNA was down-regulated only when HIF-2α was silenced, suggesting that HIF-2α is an upstream regulator of GLUT1. The HIF family of transcription factors have also been found to mediate the expression of GLUT1 in mouse ESCs, MEFs and cardiomyocytes [34], [35], [36]. However, in these cell types, it was HIF-1α, not HIF-2α which regulated GLUT1 expression. This represents a fundamental difference in the regulation of GLUT1 between mouse and human ESCs.

Using ChIP, HIF-2α was found to bind directly to the region containing a putative HRE in the proximal promoter of the GLUT1 gene only in hESCs cultured at 5% O_2_. This was an exciting finding since although GLUT1 has been extensively studied as a hypoxia inducible gene, to the best of our knowledge this is the first report of HIF-2α binding directly to GLUT1. It is therefore possible that the increased expression of GLUT1 observed may be responsible for the greater uptake of glucose into hESCs cultured at 5% O_2_. However, since many of the genes involved in glucose metabolism including hexokinase, phosphofructokinase, glyceraldehyde-3-phosphate dehydrogenase, enolase, pyruvate kinase and lactate dehydrogenase have also been shown to be regulated by environmental O_2_, alternative mechanisms of regulation remain a possibility [34], [37], [38].

### Summary

These studies demonstrate that hESCs utilise glucose as a predominant source of energy. Highly pluripotent hESCs cultured at 5% O_2_ have a low level of O_2_ consumption, consume high levels of glucose and produce large amounts of lactate. The onset of early differentiation, through the removal of FGF2 for 16 hours in hESCs cultured at 5% O_2_, or by maintaining cells at 20% O_2_ leads to a more oxidative metabolism, demonstrated by an increased consumption of O_2_ and a decreased uptake of glucose and production of lactate. The rise in glucose uptake observed under hypoxic conditions corresponds to an increased expression of *GLUT1* which is directly regulated by HIF-2α. This data provides further metabolic support for maintaining hESCs under hypoxic conditions, rather than culturing at atmospheric levels of O_2_. Finally, our data highlights the intrinsic importance of energy metabolism for hESC maintenance and may provide a novel method for the assessment of self-renewal.

## Supporting Information

Figure S1 **Typical morphology of Shef3 hESCs on day 3 post-passage cultured at 5% O_2_ (A), 5% O_2_ in the absence of FGF2 for 16 hours (5% O_2_– FGF2; B) and 20% O_2_.** Scale bar = 100 µm.
(TIF)
