# Supplementary figures and images for: Environmental Oxygen Tension Regulates the Energy Metabolism and Self-Renewal of Human Embryonic Stem Cells
Source: PLoS One. 2013 May 6;8(5):e62507. doi: 10.1371/journal.pone.0062507 (PMC3645991; doi:10.1371/journal.pone.0062507)

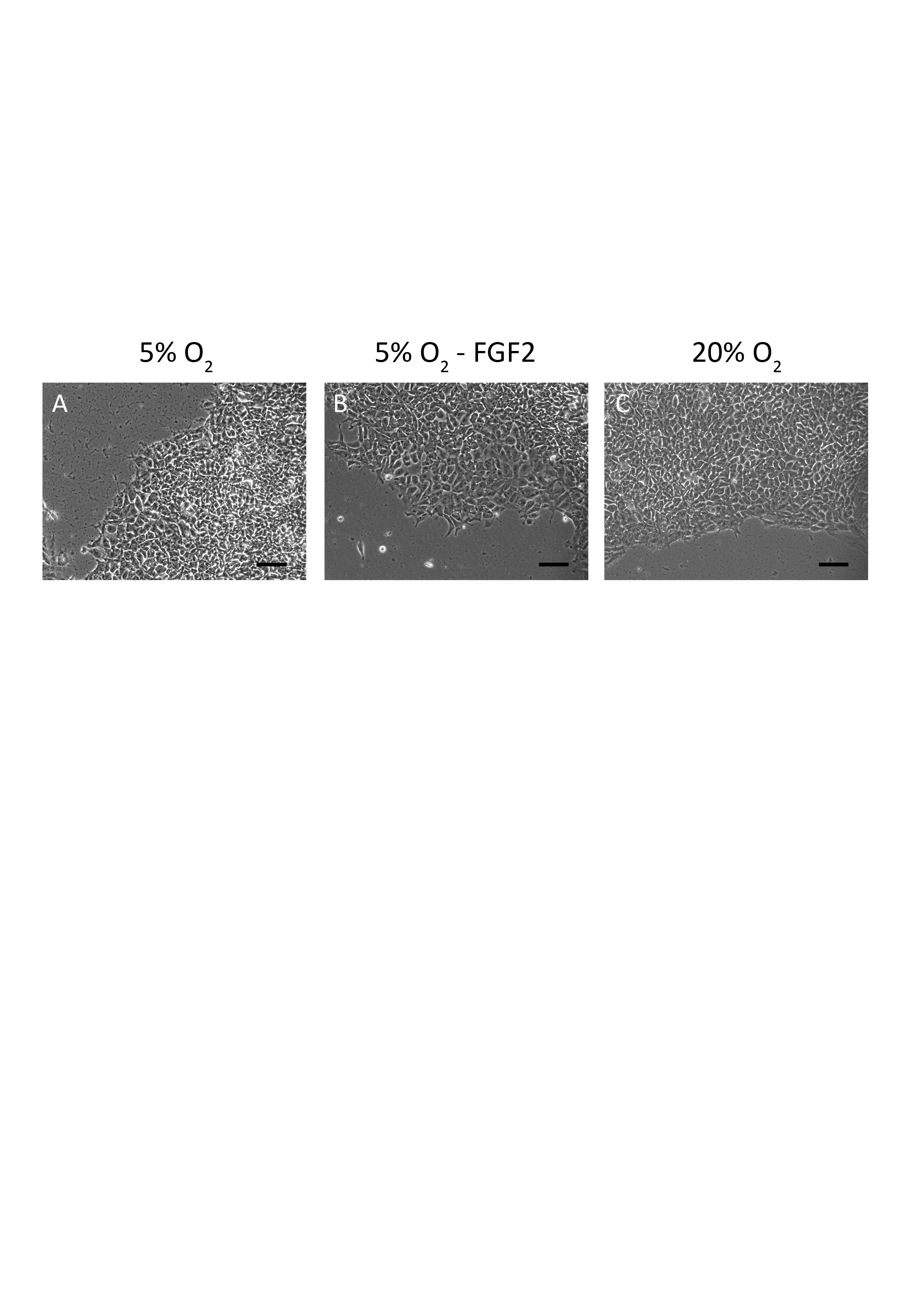

Supplement: Figure S1 — Typical morphology of Shef3 hESCs on day 3 post-passage cultured at 5% O2 (A), 5% O2 in the absence of FGF2 for 16 hours (5% O2– FGF2; B) and 20% O2. Scale bar = 100 µm. (TIF) [file pone.0062507.s001.tif]
